# Evaluation of Tumor Necrosis Factor Alpha Polymorphism Frequencies in Endometriosis 

**DOI:** 10.22074/ijfs.2015.4548

**Published:** 2015-10-31

**Authors:** Roshanak Abutorabi, Azar Baradaran, Fatemeh Sadat Mostafavi, Yasaman Zarrin, Farahnaz Mardanian

**Affiliations:** 1 Infertility Laboratory, Beheshti Hospital, School of Medicine, Isfahan University of Medical Sciences, Isfahan, Iran; 2 Department of Pathology, School of Medicine, Isfahan University of Medical Sciences, Isfahan, Iran; 3 Department of Anatomical Sciences and Molecular Biology, School of Medicine, Isfahan University of Medical Sciences, Isfahan, Iran; 4 School of Medicine, Isfahan University of Medical Sciences, Isfahan, Iran; 5 Department of Obstetrics and Gynecology, School of Medicine, Isfahan University of Medical Sciences, Isfahan, Iran

**Keywords:** Endometriosis, Tumor Necrosis Factor, Polymorphism

## Abstract

**Background:**

The pro-inflammatory cytokine, tumor necrosis factor-alpha (TNF-α), is a pathogenic element for a number of disorders. Previous studies have reported that the -1031 T/C and -238 G/A polymorphisms in the promoter region of the *TNF-α* gene are important factors in reproductive-related disorders. One of the most common gynecological diseases of women during the reproductive years is endometriosis. This study aims to assess an association between the -1031 T/C, -238 G/A and -308 G/A polymorphisms of the *TNF-α* gene promoter region to endometriosis.

**Materials and Methods:**

In this case-control study, we enrolled 65 endometriosis patients and 65 matched healthy control women by simple sampling. Polymerase chain reaction (PCR) analysis was used to analyze -1031 T/C, -238 G/A and -308 G/A polymorphisms in the *TNF-α* gene promoter region. Statistical analysis was performed using the chi-square test. P values less than 0.05 were considered statistically significant.

**Results:**

We found a strong association between the -1031 T/C polymorphism in the promoter region of the *TNF-α* gene with endometriosis (P=0.001). There were no significant associations between the -238 G/A (P=0.243) and -308 G/A (P=1) polymorphisms with endometriosis and again endometriosis stages have no association with these polymorphisms.

**Conclusion:**

The -1031 T/C polymorphism and CC genotype can be used as a relevant marker to identify women at risk of developing endometriosis.

## Introduction

Endometriosis, which is the presence of endometrial cells at ectopic sites outside the uterine cavity, is a common, complicated problem in women (1,2). Although extensive research has been performed to improve our understanding of endometriosis, its natural history remains uncertain, with an indefinite etiology, unpredictable clinical presentation, problematic diagnosis, and poorly standardized treatment. Endometriosis is a cause of morbidity attributed to pelvic pain and infertility among 15-25% of women during their reproductive years (3). The prevalence of endometriosis varies from 109 to 247 per 100,000 (4). 

Endometriosis is usually limited to the pelvis. This benign disease is characterized by peritoneal inflammation, fibrosis, adhesions, and ovarian cysts, but displays features of malignancy, such as neovascularization, local invasion, and distant metastasis (5). Hormonal, immunological, environmental, genetic factors and reflux as a mechanical factor have been implicated in its etiology but provide inconclusive explanations. More recently, an association between infectious factors and initiation of endometriosis has been proposed (3). The theory that endometrial tissue and cells reach the peritoneal cavity through retrograde menstruation along the fallopian tubes is widely conventional (6). However, although retrograde menstruation is found in 90% of women, only 10-20% of women are inflicted with endometriosis, which suggests the possibility that genetic and immunologic factors are involved (5). 

Recently, an increasing body of evidence has reported a genetic basis for endometriosis. This evidence has stimulated and motivated researches toward the genes involved in its pathogenesis. However, even with significant efforts, the exact genetic mechanisms remain unknown. Until now, numerous genes such as detoxification enzyme genes, as well as estrogen and progesterone receptor genes have been considered in relation to endometriosis (7). 

The inflammatory response in endometriosis may be mediated by proinflammatory cytokines such as tumor necrosis factor-alpha ( TNF-α ). For the first time, TNF-α has been recognized in peritoneal fluid of women with endometriosis, which introduced an association between endometriosis and disorders of the immune system (8). Study has shown that several products of the immune system such as interleukin-1 ( IL-1 ), IL-6 and TNF-α play an important role in the establishment and maintenance of endometriosis (9). Several lines of evidence support the involvement of TNF-α as an important factor in the development of inflammatory pathologies such as increased levels of TNF-α in the peritoneal fluid of women with endometriosis which is correlated with disease severity (10) and the introduction of TNF-α as a motivator of ectopic endometrial tissue implantation (11). In addition, anti-TNF therapy is an essential part of endometriosis treatment (12) and an association between *TNF-α* gene polymorphisms with endometriosis has been reported in in several ethnic populations (13). 

TNF-α is a potent immunomodulator and proinflammatory cytokine that plays an important role in the initiation and regulation of immune responses. It has been implicated in the pathogenesis of autoimmune and infectious diseases (14). TNF-α receptors such as TNFR1 and TNFR2 perform and manage its function (15). TNF-α plays a critical role in cellular proliferation, differentiation, inflammation, apoptosis, tumorigenesis and viral reproduction. Although numerous cell types produce TNF-α, it is typically produced by monocytes and macrophages (7,16). IL-1, IL-6 and TNF-α are produced by stimulated macrophages and activated leukocytes (17). TNF-α is one of the 20 genes of the HLA system which maps to chromosome 6p21.3; it spans approximately 3 kb and has 4 exons. The last exon codes for more than 80% of the secreted protein (18). 

TNF-α is also a significant source of genetic variability. Many single nucleotide polymorphisms ( SNPs ) in the promoter region of the *TNF-α* gene can play a part in the transcriptional regulation of this gene. SNPs are defined as genomic variations or differences among individuals (16). The substitution of G to A at position -238, G to A substitution at position -308, C to T substitution at position -857, C to A substitution at position -863, and the T to C substitution at position -1031 have been described in the proximal promoter of the *TNF-α* gene (19,24). For example, chronic inflammatory diseases such as rheumatoid arthritis, ulcerative colitis, and Crohn’s disease show significant correlations with -G308A and -C850T polymorphisms (25,26). In pregnant women with preeclampsia and eclampsia, a significant association with -C850T polymorphism has been observed (27). 

Although studies evaluated the association between *TNF-α* gene polymorphisms and endometriosis, their results were incompatible (28,29). Incidental positive findings, an insufficient statistical power that yielded negative results due to small study populations or genetic variation that led to heterogeneity of the study populations might explain the differences between studies (16). Therefore, additional studies from different geographical areas might shed light on the role of TNFαpolymorphisms in endometriosis. The aim of this study was to assess the associations of -1031 T/C, -238 G/A and -308 G/A polymorphisms in the *TNF-α* gene promoter region with endometriosis. 

## Materials and Methods

### Subjects and sampling

This case-control study was conducted at Beheshti Hospital, affiliated with Isfahan University of Medical Sciences, Isfahan, Iran. The study was approved by the Ethical Committee of Isfahan University of Medical Sciences. The study enrolled 130 individuals, 65 surgically confirmed cases of endometriosis and 65 healthy women who presented for normal deliveries. Control women had normal vaginal deliveries, as well as no family history, clinical symptoms, or diagnostic evidence suggestive of endometriosis. All individuals were informed about the study and signed an informed consent. The case and control groups were individually matched for age. The sample size was calculated using the proportion comparison formula by taking into consideration a confidence coefficient of 95% and statistical power of 80%. In order to participate in this study, cases had to be diagnosed with stages II, III, or IV endometriosis via a laparoscopy. Women with myoma and any benign or malignant mass were excluded from the study. A total of 2 ml of peripheral blood was collected from all participants along with clinical data, personal and family histories. 

### DNA isolation and genotype analysis

DNA was isolated from peripheral white blood cells using the PrimePrep Genomic DNA Isolation Kit (GeNet Bio, Korea) according to the manufacturer’s instructions. The DNA was stored at -20˚C until processed. Genotyping for the TNF-α polymorphism was performed by polymerase chain reaction (PCR) with specific primers, followed by restriction fragment length polymorphism (RFLP) analysis. A three-step PCR was performed using a TC-512 thermal cycler (Techne Company, UK). PCR amplification was performed in a volume of 20 μL for each sample. The reaction was done using 10 mM tris-HCl (pH=8.3), 50 mM KCl, 1.5 mM MgCl2, 0.2 mM of each dNTP, 0.4 mM of each primer (forward and reverse), one unit Taq polymerase, and 50 ng of genomic DNA. Briefly the PCR conditions included an initial denaturation at 95˚C for 5-10 minutes, followed by 35 cycles at 94˚C for 30 seconds, annealing steps that were dependent on each primer, extension steps at 72˚C for 60 seconds, and a final extension at 72˚C for 10 minutes. PCR products were digested using 1 μL of BbsI, BamHI or NcoI restriction enzymes for each sample in a volume of 10 μL. For the TNF-α -1031 polymorphism T allele, the enzyme cut the PCR product into 251 and 13 bp fragments. For the C allele 251, the PCR product was cut into 71 and 13 bp fragments. Samples that had the G allele at position -238, BamHI digestion produced 123 and 42 bp fragments. Undigested 165 bp fragment illustrated A allele. For the TNF-α -308 polymorphism G allele, the NcoI enzyme cut the PCR product into 107 and 24 bp fragments, For the A allele, the 131 bp fragment remained undigested (Table 1).

TNF-α polymorphisms were detected after separation of enzyme-treated PCR products on a 2% agarose gel, followed by GelRedTM staining.

**Table 1:** Primer sequences and PCR-RFLP analysis data from TNF-α polymorphisms


Polymorphism	Primers	PCR product	Restriction enzyme	Annealing temp.	Alleles

-1031 T/C	F 5’-TATGTGATGGACTCACCAGGT-3’	264 bp	BbsI	55˚C for 1 minute	251 bp + 13 bp (T)180 bp + 71 bp + 13 bp (C)
	R 5’-CCTCTACATGGCCCTGTCTT-3’				
-238 G/A	F 5’-AAA CAG ACC ACA GAC CTG GTC -3’	165 bp	BamHI	64˚C for 1 minute	123 bp + 42 bp (G)165 (A)
	R 5’-CTC ACA CTC CCC ATC CTC CCG GAT -3’				
-308 G/A	F 5’-GAGGCAATAGGTTTTGAGGGCCAT -3’	131 bp	NcoI	60˚C for 30 seconds	107 bp + 24 bp (G)131 (A)
	R 5’-GGGACACACAAGCATCAAG -3’				


PCR-RFLP; Polymerase chain reaction-restriction fragment length polymorphism and TNF-α; Tumor necrosis factor-alpha.

### Statistical analysis

Data are reported as mean ± SD. The chi-square test was used for statistical comparisons between group means. The odds ratio (OR) (2) and 95% confidence interval (CI) were estimated. Comparisons of the allele and genotype distributions were performed by SPSS version 19 software. P values less than 0.05 were considered significant.

## Results

In this study, we compared 130 subjects in terms of the -1031T/C, -238G/A and -308G/A polymorphisms. There were 29 ( 22.3% ) patients with stage II endometriosis, 31 ( 23.8% ) with stage III, and 5 ( 3.8% ) patients diagnosed with stage IV endometriosis. The mean age for cases was 30.53 ± 7.18 years and the mean age for controls was 29.04 ± 7.25 years, which was not statistically significant ( P=0.145 ). 

Totally, in both case and control groups, the homozygous TT genotype was seen in 84 ( 64.62% ), TC in 33 ( 25.38% ), and CC in 13 ( 10% ) blood samples for the -1031 T/C polymorphism. We observed the homozygous GG genotype in 63 ( 48.46% ), GA in 53 ( 40.77% ), and AA in 14 ( 10.77% ) blood samples for the -238G/A polymorphism. There was no evidence of any genotype related to the -308G/A polymorphism in either the patient or control group. 

**Fig.1:**
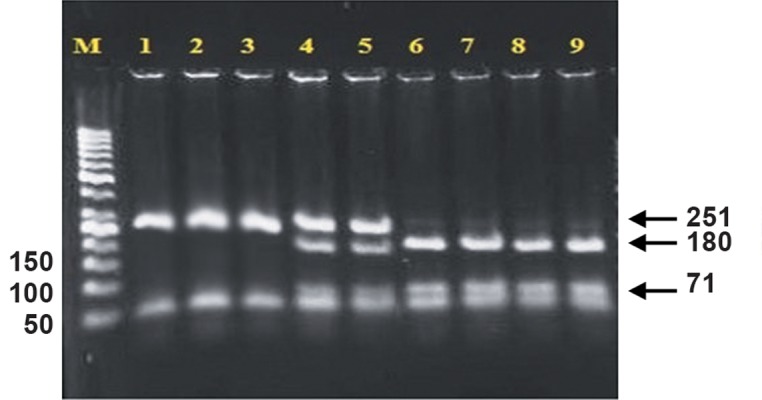
Agarose gel electrophoresis of polymerase chain reaction (PCR) assays for identification of the -1031 (T/C) single nucleotide polymorphism (SNP).

We observed significantly more of the -1031 CC genotype in the endometriosis group ( 20% ) compared with the control group ( 0%, P=0.001 ). The TT and TC genotypes were more prevalent in the control group. This difference for the TT genotype was significant ( P=0.001 ). There were fewer cases of the homozygous TT genotype in blood samples of patients with endometriosis ( 55.38% ) compared to healthy women ( 73.85% ). There were 24.65% of samples from the endometriosis group with the TC genotype compared to 26.15% of samples from the control group ( P>0.05, Fig.1 ). 

**Fig.2:**
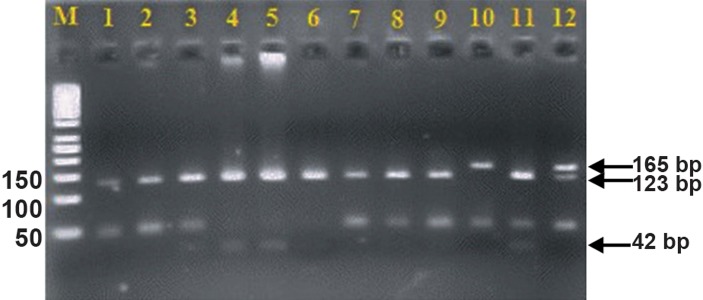
Agarose gel electrophoresis of polymerase chain reaction (PCR) assays for identification of the -238 (G/A) single nucleotide polymorphism (SNP).

Although the -238 GG genotype was less frequent in the endometriosis group ( 41.54% ) compared to the control group ( 55.38% ) and the -238 TC genotype was more frequent in the endometriosis group ( 47.7% ) compared to the control group ( 33.85% ), these differences were not significant ( P>0.05, Fig.2 ). Table 2 shows the genotype distributions of SNPs in the case and control groups. 

**Fig.3:**
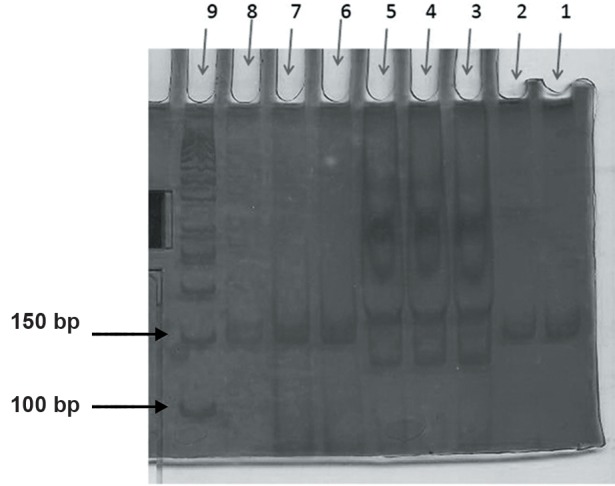
Agarose gel electrophoresis of polymerase chain reaction (PCR) assays for identification of the -308 (G/A) single nucleotide polymorphism (SNP).

In terms of the -308 polymorphism, none of the control and patient group samples contained the GA or AA genotypes. All samples from both groups were of the GG genotype (Fig .3). 

Logistic regression analysis with the backward conditional method showed that only different genotypes of the -1031 polymorphism showed significant relations with risk of endometriosis ( P<0.001 ). The risk of endometriosis in the CC genotype was significantly more than the other genotypes ( OR=0.39, 95% CI=0.22-0.7, P=0.002 ). 

The frequency of different genotypes according to the disease stage is shown in table 3. There was no significant difference in the frequency of these three polymorphisms in the *TNF-α* gene promoter region noted in relation to disease stage. 

**Table 2:** Genotype distribution of single nucleotide polymorphisms (SNPs) in the case and control groups


Genotype	Group (%)		Total	P value
	Control	Case		

-1031 T/C				
TT	48 (73.85)	36 (55.38)	84 (64.62)	0.001
TC	17 (26.15)	16 (24.65)	33 (25.38)	
CC	0	13 (20)	13 (10)	
-238 G/A				
GG	36 (55.38)	27 (41.54)	63(48.46)	0.245
GA	22 (33.85)	31 (47.7)	53 (40.77)	
AA	7 (10.77)	7 (10.77)	14 (10.77)	
-308 G/A				
GG	65 (100)	65 (100)	130 (100)	1.00
GA	0	0	0	
AA	0	0	0	


-1031 T/C (P=0.001, χ2=19.7, df=2, OR=0.39, 95% CI=0.22-0.7), -238 G/A (P=0.245, χ2=2.8, df=2), -308 G/A (P=1.00). χ2; Chi-square, OR; Odds ratio, df; Degree of freedom and CI; Confidence interval.

**Table 3:** Genotype distribution of TNF-α single nucleotide polymorphisms (SNPs) based on endometriosis stages


Genotype	Control (65)	Stage II (29)	Stage III (31)	Stage IV (5)	Total (130)	P value

-1031 T/C						
TT	48	17	15	4	84	0.42
TC	17	8	7	1	33	
CC	0	4	9	0	13	
-238 G/A						
GG	36	11	15	1	63	0.076
GA	22	17	10	4	53	
AA	7	1	6	0	14	
-308 G/A						
GG	65	29	31	5	130	1.00
GA	0	0	0	0	0	
AA	0	0	0	0	0	

-1031 T/C (P=0.42, χ2=3.9, df= 4), -238 G/A (P=0.076, χ2 = 8.5, df=4), -308 G/A (P=1.00). χ2; Chi-square, df; Degree of freedom and TNF-α; Tumor necrosis factor-alpha.

## Discussion

In this study, we investigated the association of endometriosis with three common polymorphisms in the promoter region of the *TNF-α* gene in an Iranian population according to PCR-RFLP analysis. We observed no significant differences in the frequencies of the -238 and -308 promoter polymorphisms of the *TNF-α* gene between endometriosis patients and controls. However there was a significantly lower frequency of the -1031T allele observed in patients compared to controls.

We can explain the association in different ways. For example the -1031T polymorphism may protect patients from the most severe forms of endometriosis. Also linkage disequilibrium between another gene and this polymorphism may cause this association. 

Minimal/mild endometriosis represents a normal physiological process usually found in asymptomatic women. In this study, we have performed a separate analysis on patients related to the stage of their disease. When the endometriosis cases were divided into subgroups with stages II, III, and IV disease, we observed no significant difference in the frequency of these three polymorphisms in the *TNF-α* gene promoter. 

It is well known that several molecular entities play a role in establishing and maintaining endometriosis. The relationship between TNF-α and endometriosis has been indicated in several studies making it a good candidate gene. TNF-α is produced during inflammatory processes. It is a pro-inflammatory cytokine involved in numerous infectious and inflammatory processes (30). Different researches have explained the nature of endometriosis as an inflammatory disorder (1). A number of studies assessed different TNF-α polymorphisms in endometriosis patients (13,30). 

Wilson et al. (21) emphasized the importance of several polymorphisms in the promoter region of the *TNF-α* gene. Many studies have confirmed the association between the polymorphisms at positions -238, -308, -857, -863 and -1031, increased transcriptional activity and production of TNF-α (23,31). There is also evidence for association of the -238 G/A polymorphism with insulin resistance syndrome and obesity (32), as well as an association of -308 G/A polymorphism with various inflammatory and autoimmune diseases (33). Previous studies have illustrated relations between certain immune-mediated diseases and -857, -863, -1031 polymorphisms (20,34). Two studies performed in Australia and Korea reported -238G/A and -308G/A polymorphisms in endometriosis patients but did not describe any association. Regionally and geographically, the rates of -238A and -308A alleles in TNF-α have been shown to significantly differ (35), hence there is a need for large sample sizes in order to study relationships between diseases and these polymorphisms. 

The -1031T/C polymorphism is reported to be associated with Behcet’s disease (31), Crohn’s disease (34) and Crohn’s extra-intestinal manifestations that include uveitis, erythema nodosum and large joint arthropathy (36). Studies have shown increased frequency of the -1031T allele in patients with hyperandrogenism and in those with ulcerative colitis (34,37). In contrast, a lower frequency of the -1031T allele is reported in patients with Crohn’s and Behcet’s diseases compared to controls (31,34). 

The findings of this study were in concordance with the outcomes of studies by Ahmad et al. (31) and Negoro et al. (34) who reported a low frequency of the -1031T allele in the mentioned diseases compared to normal population, but this polymorphism had no significant correlation with other disorders. In order to describe the importance of the TNF-α -1031T/C polymorphism, additional research would be necessary to clarify the relationship between this multi-functional proinflammatory cytokine with endometriosis. 

In the current study, differences in the frequency of the -1031T/C alleles was not associated with the severity of endometriosis. Asghar et al. (16) reported that a decreased frequency of the -1031C allele was related to severe endometriosis but they did not find an association with less severe disease. The low number of patients in the current study, in particular the severe forms of endometriosis could explain this difference. The results implied that the T allele could provide protection from endometriosis. 

Our data showed an increase in the frequency of the genotypes -1031 C/C and -238 G/A as well as a decrease in the frequency of the -1031 T/T,T/C and -238 G/G genotypes in patients with endometriosis. However only the -1031 T/T,C/C polymorphism changes were significant. There was no association between the stage of endometriosis and different genotypes. 

Although there was no association between the TNF-α polymorphism and endometriosis in the majority of similar studies (38,39), we did find associations reported in some studies. In a study performed on Indian women, there was a significant increase of endometriosis in women carriers of the -850 T/T genotype and TT genotype increased the risk of endometriosis. In the Japanese population, TNF-α -1031T/C polymorphism was associated with reduction of endometriosis risk (39). In the Korean and Japanese populations, advanced stage endometriosis was seen mostly in patients who had the TNF-α -1031T/C polymorphism (13,30) but there were no significant differences in frequencies between endometriosis cases and controls for TNF-α -238G/A, -308G/A, -857C/T, and -863C/A polymorphisms (13). TNF-α polymorphisms showed no significant association with endometriosis in Australian, Chinese, Taiwanese, and Austrian populations (1). Asghar et al. (16) assessed the -238C/T polymorphism of TNF-α with inflammatory diseases and endometriosis. However, most did not report any significant associations with endometriosis. 

Elevated TNF-α levels in peritoneal fluid have been associated with up-regulated TNF-α production in peritoneal macrophages and peripheral monocytes of women with endometriosis (11). The functional role of TNF-α in endometrial tissue is unknown. It has been assumed that a TNF-α polymorphism that alters its transcription/expression subsequently enhances its level, leading to increased proliferation and decreased apoptosis as seen in the inflammatory cascade. 

The results of a recent meta-analysis study implies that TNF-α -1031C is associated with a higher risk of endometriosis in Asian individuals in homozygote comparisons and the recessive genetic model (16). However in another study the TNF-α -1031T/C polymorphism has shown a relation with endometriosis in the Iranian population. They reported that the -1301C allele might have a protective role in the development of endometriosis (40). In this study, an association was demonstrated between the -1031C/T TNF-α polymorphism and endometriosis, which indicated that it could be used as a relevant molecular marker to assess the risk of endometriosis. 

Due to the small number of studies (39,40) that have focused on *TNF-α* gene polymorphisms and the limited number of cases and controls in these studies, it is necessary to perform additional studies with larger sample sizes and well-matched controls stratified by stage, ethnicity, or other risk factors. These studies may assist in explaining the possible roles of the *TNF-α* gene polymorphisms in endometriosis, particularly in other ethnic populations. 

## Conclusion

The TNF-α promoter -1031 T/C polymorphism is associated with decreased risk of endometriosis in an Iranian population. Our data have demonstrated decreased frequency of the -1031T polymorphism in the promoter region of the *TNF-α* gene in the most severe cases of endometriosis in our studied population. This finding has suggested that the -1031T polymorphism may play a protective role in endometriosis progression. Although this model is biologically acceptable, we recognize that our conclusions are based on relatively small numbers and require confirmation from additional independent studies. 
